# A Young Patient with Painful Penile Lesions

**DOI:** 10.7759/cureus.6397

**Published:** 2019-12-16

**Authors:** Christopher Gaeta, Stephen Scholand, Brandon Blakey, Richard Pescatore

**Affiliations:** 1 Emergency Medicine, Children's Hospital of Philadelphia, Philadelphia, USA; 2 Internal Medicine: Infectious Diseases, University of Arizona, Tuscon, USA; 3 Emergency Medicine, Crozer Keystone Health System, Chester, USA; 4 Emergency Medicine, Crozer-Keystone Health System, Chester, USA

**Keywords:** chancroid, differential, infectious diseases, medical education, case study, case report

## Abstract

Chancroid is a rare infection in the United States and many other developed countries. Infrequently identified as a cause of genital ulcer disease, chancroid’s atypical presentation has only been reported in approximately 20 cases annually in the United States since 2011. Infection with the causative organism, Haemophilus ducreyi, leads to an erythematous papule that rapidly evolves into a pustule. Infected individuals commonly have more than one ulcer about 2 cm in diameter that is typically noted as painful. The base of the ulcer is usually covered with a gray or yellow purulent exudate and bleeds when scraped.

Despite a heavy focus in preclinical medical education, the notably rare chance to see and diagnose chancroid in clinical practice adds to the complicated profile of this infection’s identification and subsequent treatment. Such lack of familiarity contributes to reports of accuracy of clinical diagnosis ranging from 30% to 80%.

## Introduction

The following report presents a challenge shared by a vast majority of providers in the accurate diagnosis of chancroid. The rare clinical presentation of a patient with painful genital lesions with negative lab results for the typical array of sexually transmitted disease poses a challenge for a provider that ultimately requires a team-based approach that (in nearly all cases of chancroid) relies on clinical findings rather than awaiting highly specialized and costly laboratory testing to confirm the diagnosis.

Infection with the causative organism, *Haemophilus ducreyi*, leads to an erythematous papule that rapidly evolves into a pustule. Infected individuals commonly have more than one ulcer about 2 cm in diameter that is typically noted as painful. The base of the ulcer is usually covered with a gray or yellow purulent exudate and bleeds when scraped.

Despite a heavy focus in preclinical medical education, the notably rare chance to see and diagnose chancroid in clinical practice adds to the complicated profile of this infection’s identification and subsequent treatment. Such lack of familiarity contributes to reports of the accuracy of clinical diagnosis ranging from 30% to 80%.

The difficulties of accurately diagnosing the infection begin with the rarity of its presentation. More specifically, roughly about 20 cases on an annual basis have been reported by providers in the United States in the past two decades. The added barriers to obtaining a confirmed diagnosis are unrealistic to many providers. This is directly due to the absence of rapid and widespread availability for testing of chancroid. The highly specialized nature of a definitive test for chancroid is a means of diagnosis not within a realistic means of access for an overwhelming majority of providers [1-2]. As such, Center for Disease Control and Prevention (CDC) guidelines and clinical practice provide for presumptive diagnosis and treatment in the correct clinical setting [3-4]. 

## Case presentation

**Figure 1:**
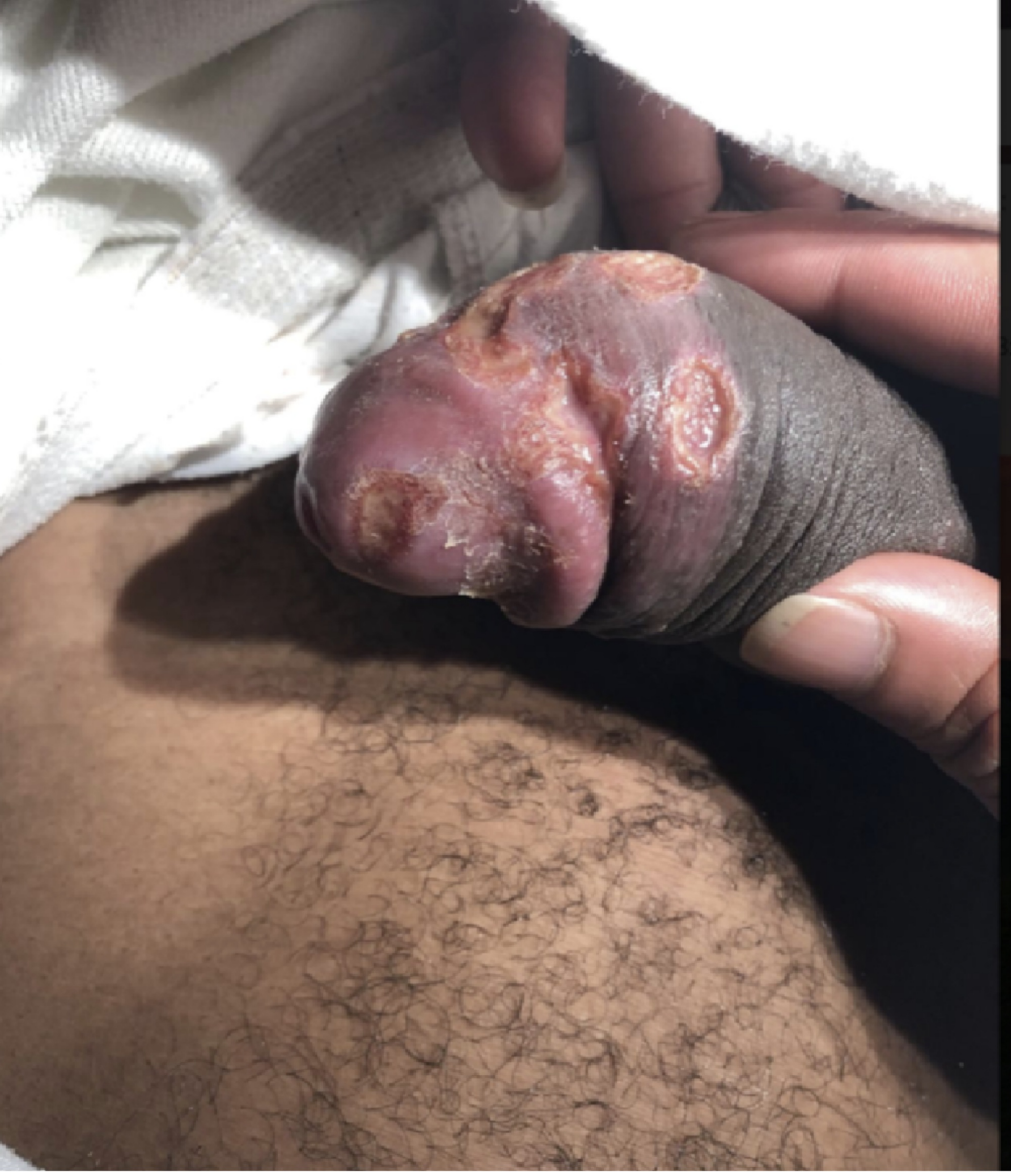
Lesions on the glans and distal penile shaft.

A 29-year-old male presented to the ED with painful penile lesions that had progressed for approximately five weeks prior to presentation. He had recently been released from a correctional facility. The patient reported unprotected sex with multiple male and female partners. The patient stated that the lesions began as small erythematous macules that slowly progressed to ulcers. Urine polymerase chain reaction (PCR) for gonorrhea and chlamydia, fourth generation HIV testing, rapid plasma regain (RPR) screening for syphilis, and herpes simplex virus (HSV) DNA testing of the lesions were all negative. The ulcers were cultured; however, special media for H. ducreyi were not available at our institution and standard culture techniques grew only *Staphylococcus epidermidis* [5] (Figure 1).

 

## Discussion

The patient was treated for a diagnosis of probable chancroid with 250 mg IM ceftriaxone and 1000 mg oral azithromycin. At three-week follow-up, the lesions had nearly resolved compared to the initial presentation several weeks prior. This resolution is consistent with the expected course, as in cases of chancroid, clinical improvement usually occurs promptly after treatment is initiated.

Relief of pain is noted by most patients within two to three days, and objective improvement in ulcers is usually apparent within a week. Both of these were consistent with the patient's progress after his first three weeks of treatment initiation. Patients with chancroid should abstain from sex until ulcers have dried/resolved, and condom use should be stressed [6]. 

Providers should be aware of the presentation of chancroid in individuals who are confirmed to not have more typical sexually transmitted diseases. Scarce diagnostic media to diagnose chancroid in a laboratory should be considered as noted in the introduction. This added challenge to a provider suspecting chancroid as a diagnosis is augmented by limited educational opportunities to expose a case to providers in training. Therefore, as followed in this particular case report, clinicians should particularly begin to consider chancroid in patients with genital ulcers after more common sexually transmitted diseases such as HSV or syphilis is investigated based on the case presentation [7-8].

**Figure 2:**
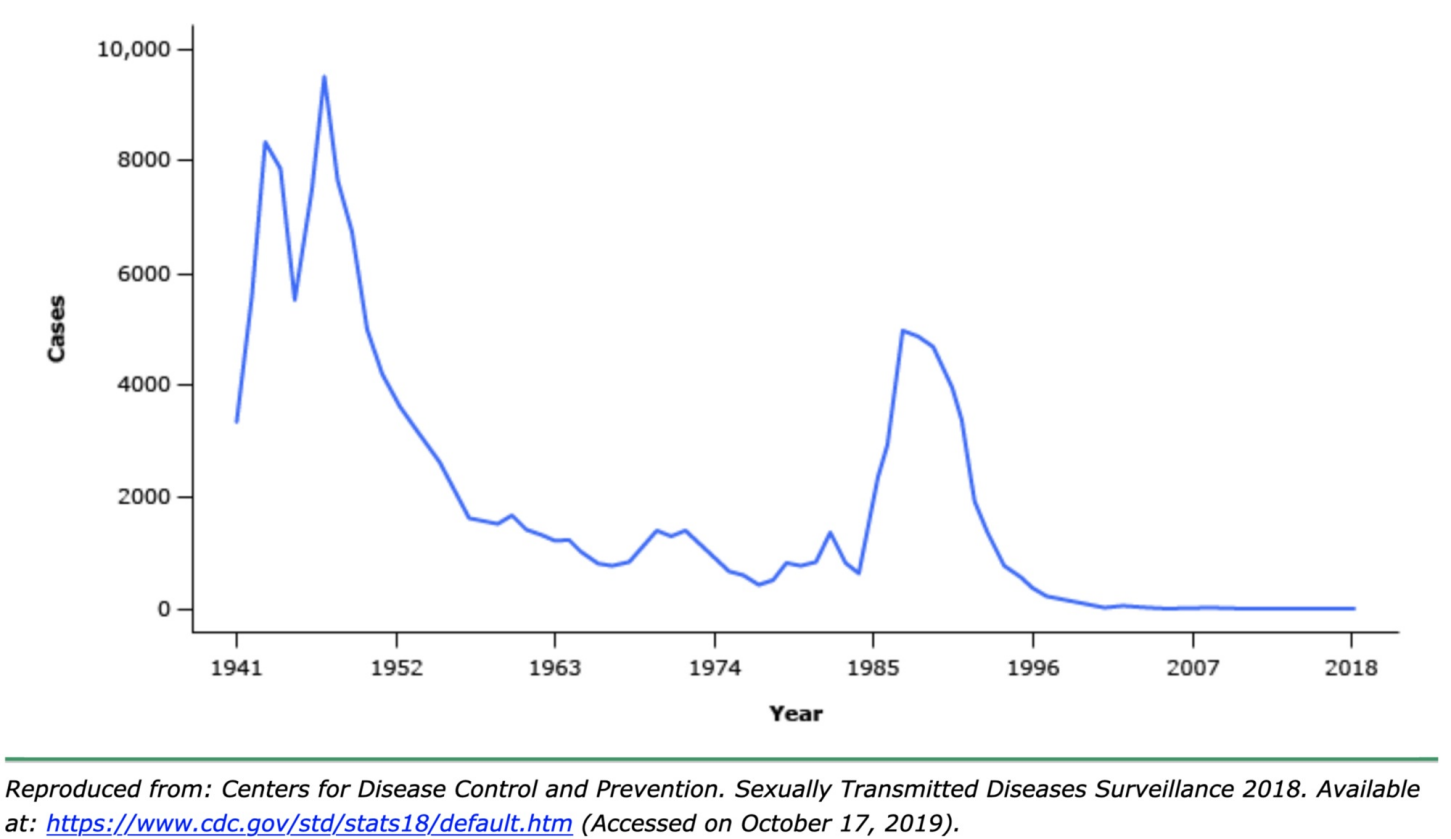
CDC report, prevalence of chancroid in the United States in the years, 1941-2018. CDC, Center for Disease Control and Prevention

Underscoring the educational significance of this case, CDC data dating back to 1941 reinforces the rare presentation of chancroid on a national scale (Figure 2).

In past decades when chancroid has appeared, transmission has been typically moderate to large and localized [4]. As such, it is critical for clinicians to recognize this relatively rare clinical entity and administer presumptive treatment, especially as confirmatory testing is unlikely to be obtainable.

## Conclusions

This case highlights the challenges that providers face in recognizing and diagnosing chancroid. Perhaps the most significant challenge is accurately diagnosing chancroid rests with the inability to obtain confirmatory microbiological testing. Laboratories with the ability to test for *H. ducreyi* via special culture media are not only highly specialized and costly but also scarce in the United States and abroad. This obstacle has made it difficult to estimate the true incidence rate. As such, providers rely on clinical criteria and differential diagnosis to confirm the presentation of chancroid. 
